# Inferring Trajectories of Psychotic Disorders Using Dynamic Causal Modeling

**DOI:** 10.5334/cpsy.94

**Published:** 2023-08-28

**Authors:** Jingwen Jin, Peter Zeidman, Karl J. Friston, Roman Kotov

**Affiliations:** 1 Department of Psychology, The University of Hong Kong, Hong Kong SAR, China; 2 State Key Laboratory of Brain and Cognitive Sciences, The University of Hong Kong, Hong Kong SAR, China; 3 Wellcome Centre for Human Neuroimaging, University College London, UK; 4 Department of Psychiatry, Renaissance School of Medicine, Stony Brook University, USA

**Keywords:** Dynamic Causal Modeling, Psychotic, Nosology, Symptom trajectory, Longitudinal model

## Abstract

**Introduction:**

Illness course plays a crucial role in delineating psychiatric disorders. However, existing nosologies consider only its most basic features (e.g., symptom sequence, duration). We developed a Dynamic Causal Model (DCM) that characterizes course patterns more fully using dense timeseries data. This foundational study introduces the new modeling approach and evaluates its validity using empirical and simulated data.

**Methods:**

A three-level DCM was constructed to model how latent dynamics produce symptoms of depression, mania, and psychosis. This model was fit to symptom scores of nine patients collected prospectively over four years, following first hospitalization. Simulated subjects based on these empirical data were used to evaluate model parameters at the subject-level. At the group-level, we tested the accuracy with which the DCM can estimate the latent course patterns using Parametric Empirical Bayes (PEB) and leave-one-out cross-validation.

**Results:**

Analyses of empirical data showed that DCM accurately captured symptom trajectories for all nine subjects. Simulation results showed that parameters could be estimated accurately (correlations between generative and estimated parameters >= 0.76). Moreover, the model could distinguish different latent course patterns, with PEB correctly assigning simulated patients for eight of nine course patterns. When testing any pair of two specific course patterns using leave-one-out cross-validation, 30 out of 36 pairs showed a moderate or high out-of-samples correlation between the true group-membership and the estimated group-membership values.

**Conclusion:**

DCM has been widely used in neuroscience to infer latent neuronal processes from neuroimaging data. Our findings highlight the potential of adopting this methodology for modeling symptom trajectories to explicate nosologic entities, temporal patterns that define them, and facilitate personalized treatment.

## Introduction

Existing psychiatric nosologies—Diagnostic and Statistical Manual of Mental Disorders (DSM) and International Classification of Diseases (ICD)—are based primarily on clinical observations. To-date, no categorical diagnosis has been developed based on the statistical modeling of how psychopathology manifests in clinical measurements over time (Krueger et al., 2018). Accordingly, current nosologies are heuristic rather than mechanistic descriptions. Many have argued that the limited validity of the ensuing classifications contributes to the sluggish pace of discovery in psychiatry (Cuthbert & Insel, 2013; Gordon & Redish, 2016; Hyman, 2010). Psychiatric diagnosis also offers limited guidance to care. Clinicians frequently forego a formal diagnostic assessment and prescribe treatment based more on symptoms than diagnosis (Taylor, 2016; Waszczuk et al., 2017), as many perceive diagnostic manuals to be unhelpful in prognostication and treatment selection (First et al., 2018).

Computational psychiatry offers to replace heuristic diagnoses with formal statistical models that can predict illness course and treatment response (Friston et al., 2017; Krystal et al., 2017; Nelson et al., 2017; Wang & Krystal, 2014). The aspiration here is that modeling symptom patterns over time can reveal underlying dynamics—and forecast clinical trajectories in the same spirit as weather forecasts. These latent (i.e., unobservable) dynamics may differ between individuals in type (e.g., whether psychosis is limited to mood episodes or occurs independent of mood disturbance) or continuous gradation (e.g., propensity to depression). Accordingly, a computational nosology would be a system based on individual variations in underlying dynamic processes that generate symptoms, and diagnosis would be the process of describing patient’s clinical course in terms of these processes, that is, what trajectory the illness follows. Course typologies play an important role in psychiatry (Angst et al., 2003; Ciompi, 1980), but they have been derived by clinical observation, and statistical modeling offers to improve their validity. Development of such dynamic models requires intensive longitudinal data with dozens, perhaps even hundreds of observations per patient (Beltz & Gates, 2017). Electronic health records, mobile monitoring, and experience sampling technologies are currently making such data accessible (Wright & Woods, 2020).

Multiple approaches are available for analysis of intensive longitudinal data. The most common strategy is modeling of trajectories defined by intercept and slope (Duncan et al., 2011; Nagin & Odgers, 2010). However, these models preclude the nonlinear dynamics that underwrite symptoms. Another strategy is to model a network of symptoms for each individual (Asparouhov et al., 2018; Epskamp et al., 2018; Fisher et al., 2017; Lane et al., 2019). This approach is truly personalized and can describe complex dynamics. However, it is focused on the level of relations among individual symptoms and does not seek to characterize constructs underpinning multiple symptoms. Identification of latent constructs may offer a more parsimonious account of the phenomena than mapping of symptom relations. Furthermore, network modeling describes the structure of relationships among its elements and is not typically concerned with forecasting.

Dynamic Causal Modeling (DCM) is an approach to modeling time series data that was developed in neuroscience and has helped to understand many neuronal phenomena (Daunizeau et al., 2011; Frässle et al., 2021; Friston et al., 2003; Papadopoulou et al., 2017). It models latent variables generating observed data and characterizes their dynamics in terms of model parameters, such as strength of causal links or directed connectivity, rate constants, time constants, and so on. DCM relies on the construction of a *generative model* that describes latent processes that give rise to observed time-series data. Here, we use a specific type of generative model, namely, state-space models, which have been widely applied in neuroscience. In neuroimaging, DCM has been used to examine complex, non-linear, and context-sensitive neuronal dynamics and connectivity across brain regions (Friston, Harrison, & Penny, 2003). This was achieved by modeling how neural activity and connectivity are disturbed by external stimulation, typically in an experimental task, which gives rise to the measurable data, e.g., the blood-oxygen-level-dependent signal in functional magnetic resonance imaging. This modeling approach has produced a large scientific literature since its development and is considered as a standard method in neuroimaging research (Daunizeau et al., 2011). The fundamental advantage of a generative (state space) model of timeseries data is that it can be used for prediction and forecasting. Perhaps the best example of this is the recent application of DCM to agent-based modeling and quantitative epidemiology (Friston et al., 2020; Gandolfi et al., 2021), affording an empirically informed approach to situational awareness, forecasting and scenario modeling of the spread of infectious diseases (Friston et al., 2020).

Applied to psychiatric symptoms, this means that the symptoms at a given time point are regarded as the observable consequence of underlying disease dynamics, which can be used to forecast change in symptoms over time and in response to specific exposures or interventions. Importantly, individual differences in these underlying dynamics (i.e., time-invariant model parameters that differ between people) can inform nosology. When provided with the symptoms, one can invert the model; that is, estimate latent disease processes from observed symptoms timeseries. Following this, the evidence for alternative candidate models can be compared: for example, to assess whether the data of a new patient, with an unknown etiology, are best explained by a model previously trained on patients with one disease relative to another. In this context, the *evidence* (a.k.a., marginal likelihood) for a model has a specific meaning: it is the probability of observing the data given the model. Testing hypotheses based on this quantity is referred to as *Bayesian model comparison*. Accordingly, DCM offers the necessary tools to explain and predict illness time course, which could, in principle, provide a new quantitative tool for computational nosology.

The current paper takes the first step towards applying DCM to psychiatric nosology. We introduce a proof-of-concept model that demonstrates the feasibility of modeling longitudinal psychiatric symptom data, using technologies that are well established in neurophysiology—and are currently being applied in public health and epidemiology. We focused on psychosis, depression, and mania symptoms, as the nosology of psychotic disorders is, arguably, unresolved, and temporal relations among symptoms are central to delineation of these constructs (Kotov et al., 2022; Kotov et al., 2013).

In what follows, we first describe a generative model for timeseries data in a psychiatric setting. Second, we illustrate fitting this model to data from a sample of patients with first-admission psychosis to evaluate its ability to explain diverse clinical (symptomatic) trajectories. Third, we used simulations to evaluate the accuracy of inferring model parameters and classifying people according to certain mixtures of parameters that give rise to distinct kinds of trajectories. Finally, we discuss the potential utility of the current approach for future nosology research.

## Methods and Materials

### The generative model

**Figure 1:**
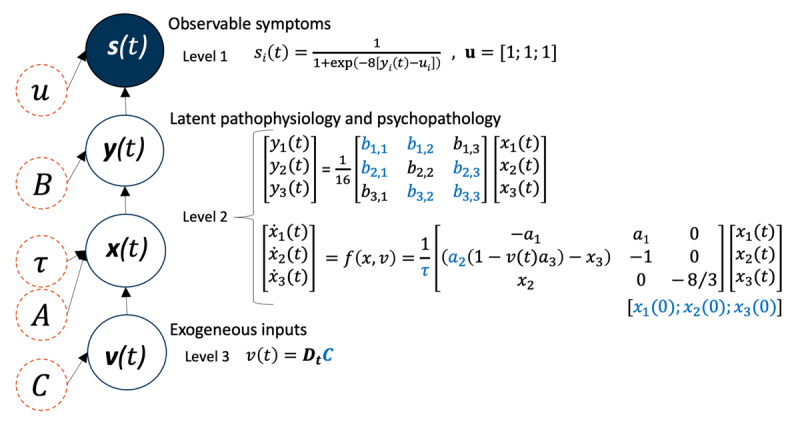
**The graphical model and definition of dynamics.** Orange empty circles (dashed) denote time-invariant parameters; deep blue empty circles (solid) denote time-variant variables whereas the deep blue filled circle denotes a time-variant observable/measurable variable; letters in light blue denote parameters whose values are allowed to vary in the current study. Level 3 models exogenous inputs, such as stressors, *v(t*) at time *t*. This is defined as a general linear model with a design matrix ***D*** (fixed) encoding a discrete cosine transform (DCT) basis set, with associated parameters ***C***. Here we use the notation ***Dt*** to indicate row *t* of matrix ***D***. Level 2 models the dynamics of pathophysiology *x(t*) as a Lorenz attractor (a set of 3 differential equations) with parameters ***A***. A linear transform yields psychopathology *y(t*) parameterized by ***A***. Finally, level 1 transforms psychopathology to observed symptoms *s(t*) in the range [0 1] which was the unit of the actual measurements.

A dynamic causal model was constructed based on a previous theoretical proposal (Friston et al., 2017) with significant modifications. Figure 1 illustrates the graphical model that specifies how a timeseries of symptoms ***s***(*t*)—that constitute observable outcomes—are generated by latent “psychopathology” states ***y***(*t*), which are themselves determined by a linear combination of “pathophysiology” states ***x***(*t*), under the influences of exogenous inputs ***v***(*t*). Also see Table 1 for a glossary of terms. Following the nomenclature of Friston et al. (2017) we refer the two levels of latent states in terms of “pathophysiology” and “psychopathology.” However, we put quotation marks around these terms because these are abstractions that do not commit to specific psychopathological constructs or pathophysiologic processes, at this stage.

**Table 1:** Glossary.


SYMBOL	INTERPRETATION

***s***(*t*)	A vector [*s*_1_(*t*); *s*_2_ (*t*); *s*_3_(*t*)] indicating time-variant symptom scores that are bounded between 0 and 1: *s*_1_(*t*)- psychosis, *s*_2_ (*t*)– depression, *s*_3_(*t*) – mania. The transformation from ***y*** to ***s*** is parameterized by ***u***.

***y***(*t*)	A vector [*y*_1_(*t*); *y*_2_ (*t*); *y*_3_(*t*)] indicating the time-variant latent psychopathology. The mapping from ***x*** to ***y*** is parameterized by ***B***.

***x***(*t*)	A vector [*x*_1_(*t*); *x*_2_ (*t*); *x*_3_(*t*)] indicating the latent time-variant pathophysiology state. Values at each time point are determined by the Lorenz system of differential equations (Figure 1). Parameterized by ***A*** and *τ*.

***v***(*t*)	A 1-by-T vector. Exogeneous inputs. Output of a DCT.

***T***	A time-invariant scalar. The number of assessment points. Determined by the number of available data points in the empirical or simulated data.

***A***	A time-invariant vector [*a*_1_; *a*_2_; *a*_3_]. Lorenz attractor parameters. In the current paper, *a*_1_= 10, *a*_3_ = 1. Only *a*_2_, namely the Rayleigh parameter, is a free parameter.

***B***	A time-invariant 3-by-3 matrix. The weights of the linear combination, mapping from ***x*** to ***y***. In the current paper, *b*_1,3_, *b*_2,2_, *b*_3,1_ were fixed at 0.

***C***	A 1-by-8 vector. Coefficients of the DCT parameterizing exogenous inputs.

***D***	The design matrix of the cosine discrete function of time.

***x***	A time-invariant vector [*x*_1_(0); *x*_2_ (0); *x*_3_(0)], indicating the initial values of the pathophysiology states.

***u***	A time-invariant vector [*u*_1_; *u*_2_; *u*_3_]. Thresholding the symptom scores from psychopathology. In the current paper, they are fixed at 1 (i.e., *u*_1_ = *u*_2_ = *u*_3_ = 1).

*τ*	A time-invariant scalar. Time constant of dynamics.


**Figure 2:**
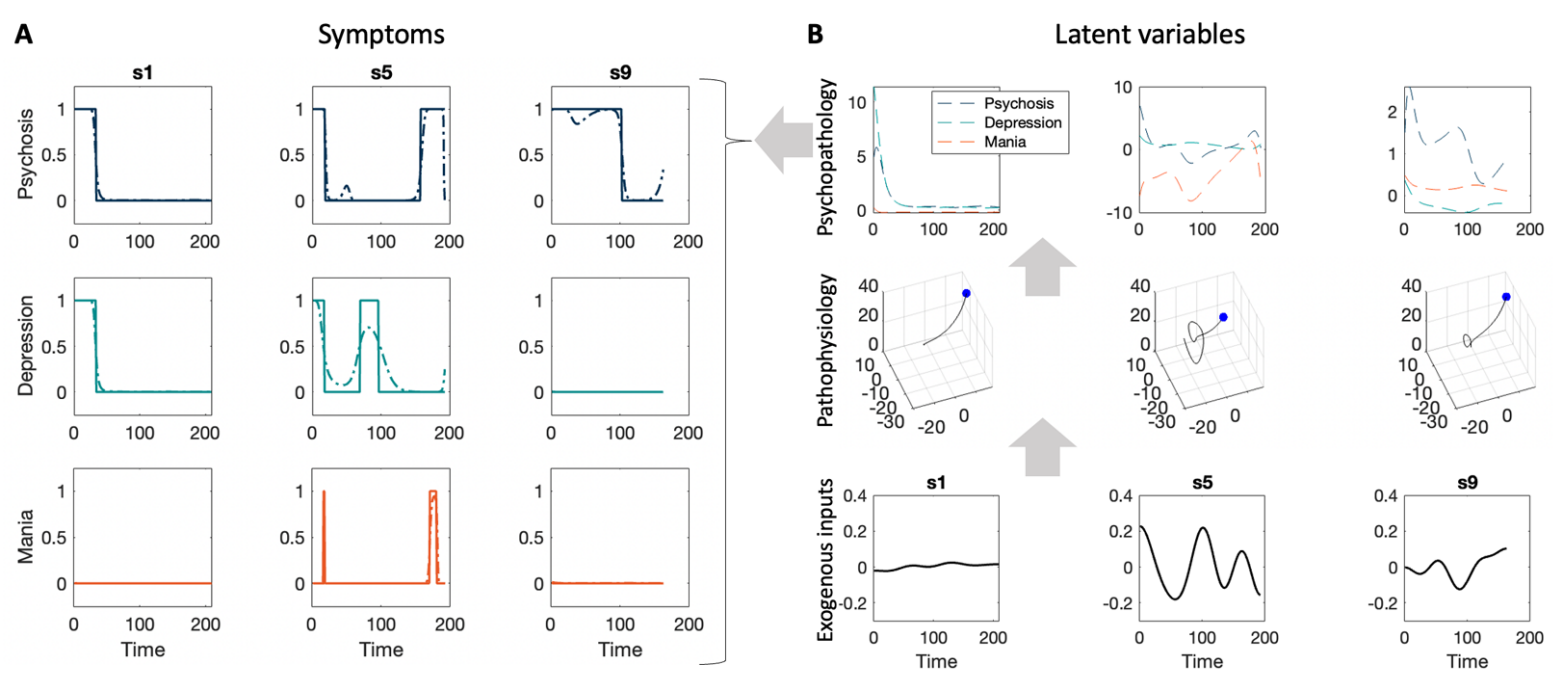
**Model fitting results. A. Estimated and actual symptom scores.** The estimated symptoms (dashed) overlaid on the empirical symptoms (solid) for three subjects. Each row corresponds to a different kind of symptom score (psychosis, depression and mania). **B. Estimated latent variables.** For the same three subjects, the estimated exogeneous inputs, the pathophysiology trajectory (the Lorenz attractor) and the psychopathology are plotted from bottom to top. For the pathophysiology state, the blue dot marks the initial state of the trajectory in the state space defined by (*x_1_,x_2_,x_3_*).

Central to this model is the concept of an abstract *space* of pathophysiology. At any given time *t*, the patient occupies a position in this space, encoded by the vector of coordinates ***x***(*t*). As the disease progresses, the patient follows a trajectory through the pathophysiology space, where the course of this trajectory is estimated from the data. The patient’s position in pathophysiology space ***x***(*t*) determines their level of psychopathology, ***y***(*t*), which is effectively binarized to generate observed symptom scores, ***s***(*t*). Therefore, in this model, symptoms are treated as a consequence of the underlying pathophysiology and resultant psychopathology.

In detail, the trajectory of the latent pathophysiology states is modelled with a Lorenz attractor with three parameters (***A***). The Lorenz attractor was chosen because it is the most simple and generic formulation of itinerant dynamics – i.e., it is a *normal form* for a system of dimension three (Shil’Nikov et al., 1993). Having three states is the minimum necessary for any system to show itinerancy or chaos. Since its introduction in atmospheric dynamics, it has been used widely for modeling chaotic and dynamic systems in physics and biology. Importantly, the Lorenz attractor can model cycles or orbits, which we anticipate will be important for capturing relapse in psychiatric illness. For demonstration purposes, for the Lorenz attractor parameters, only the 2^nd^ parameter (known as the Rayleigh parameter) was allowed to vary in the current study. We chose the Rayleigh parameter because it determines the cyclicity of the latent pathophysiological states, where cycle patterns of disease progression are of particular interest to psychiatric research (technically, changing this parameter induces bifurcations that changes the trajectory qualitatively; from a simple decay to a fixed point, through periodic cycles to chaotic dynamics). The exogenous inputs (e.g., stressors or therapeutic interventions) ***v***(*t*) are assumed to be smooth and were modeled as a discrete cosine transformation (DCT) basis set, which form the columns of a general linear model (i.e., design matrix) ***D*** weighted by parameter vector ***C***. This relatively unconstrained basis set was selected because our exemplar empirical data did not contain a detailed assessment of exogenous inputs, which could be positive (e.g., intervention) or negative (e.g., life stressor). A DCT basis set can model any smooth continuous function and is the basis set of choice, given its well-known boundary and compression properties. An 8-order DCT was chosen given the natural time constants of putative stressors and therapeutic interventions (as set out in Table 1). In practice the optimal number of basis functions could be selected for a particular application through Bayesian model comparison. A greater number of basis functions effectively allow for ‘faster’ exogenous effects. Alternatively, the DCT basis set could be replaced with regressors (dummy variables) encoding known or hypothesised exogenous inputs, although these variables were not available here. Conceptually, this models a situation where the state-space of pathophysiology has its own (autonomous) dynamics, and the exogenous inputs can be various kinds of life events, such as stressors or interventions. A linear combination of pathophysiology states (parameterized by ***B***) then yields the latent psychopathology. Our model makes the simplifying assumption that all the interesting dynamics of disease progression unfold at the level of the brain (pathophysiology), and that psychopathology and symptoms are consequences of that underlying pathophysiology. It was natural, therefore, to formulate the psychopathology as a (linear) mixture of the underlying pathophysiology states. Of course, a more involved non-linear mapping from neural dynamics to psychopathology could be entertained when comparing more fine-grained and mechanistic DCMs in the future. Finally, the latent psychopathology is transformed to measurable/observable symptoms through a logistic function parameterized by ***u***.

The priors over the parameters were specified as a multivariate normal density. As is general practice in dynamic causal modeling, these priors are relatively uninformative; thereby enabling (i) the data to constrain posterior estimates and (ii) the DCM to explain of a wide range of trajectories. A key aspect of these priors is that they generally enforce positivity through the use of lognormal priors; in other words, for example, rate and time constants are modelled as log constants, with a normal or Gaussian prior. This means the prior variance constrains proportional variation, where uninformative priors would allow changes over an order of magnitude; and tight priors allow changes from prior expectations of a few percent. See the **Supplementary materials** for MATLAB code implementing this DCM structure, including the specification of the priors (in which the prior expectation of the parameters is specified in the MATLAB variable pE and the prior covariance matrix is specified in variable pC).

### Empirical data

Data for this study were derived from the Suffolk County Mental Health Project, an epidemiologic study of first-admission psychosis (Bromet et al., 1992; Kotov, Fochtmann, et al., 2017). Patients were recruited from the 12 psychiatric inpatient units of Suffolk County, NY, between 1989 and 1995. Inclusion criteria were first admission either current or within six months, clinical evidence of psychosis, ages 15–60 years old, IQ > 70, proficiency with English, and no apparent general medical etiology. The study was approved annually by the institutional review boards of Stony Brook University and the participating hospitals.

Face-to-face and phone assessments were conducted by master’s level mental health professionals every three months. Medical records and interviews with significant others were also obtained throughout the follow-up. These detailed data allowed raters to chart symptom course for 48 months following first admission (Kotov et al., 2013). This furnished ratings for severity of psychosis, mania, and depression (0 = no symptoms, 1 = subthreshold or full symptoms) in every week of the interval. As the goal of the current paper was to establish the methodology—rather than answering a clinical question—only a subset of subjects (N = 10) was selected (randomly) from this study. After excluding one subject with a limited number of assessments (< 50 available data points), nine subjects were included in the subsequent analyses. Analyzing data from these nine subjects allowed us to both capture diverse clinical trajectories and allowed us to examine each subject individually. These subjects were selected to represent a variety of distinct clinical trajectories typically seen in psychotic disorders. All patients experienced a psychotic episode, which was brief for five cases, recurring for one case, and chronic for three cases. Depressive episodes were observed in six cases and absent in three cases. Manic episodes were present in five cases and absent in four cases. Psychosis was limited to mood episodes in five cases and occurred outside mood episodes in four cases. Please see **Supplementary Figure 1**.

### Dynamic causal modeling (DCM)

#### Demonstrating model flexibility

We first ensured that the model could explain various types of clinical trajectories, demonstrating its flexibility. To this end, we conducted model-fitting (model inversion) using nine subjects’ empirical data. Model inversion entails finding the posterior probability distribution over the model parameters using standard variational Bayes methods, given each subject’s data [specifically, we used the Variational Laplace model estimation scheme: (Friston et al., 2007; Zeidman et al., 2022)]. The parameters that were allowed to vary—to explain between subject variations in clinical trajectories—are written in blue text in Figure 1 (also see Table 1). In brief, the parameters that were allowed to vary were (i) the key parameter governing pathophysiological dynamics (the Rayleigh parameter, *a*_2_), (ii) its starting values, *x*(0), (iii) the mapping from pathophysiological to psychopathological states, *b*, (iv) the overall time or rate constants of pathological changes *τ*, and finally (v) the variable influencing the exogenous inputs, but this variable is not of particular interest in the current study.

There were two special aspects to this model fitting. First, because we are dealing with a highly nonlinear system, we had to account for local maxima during model inversion. This was addressed using a multi-start algorithm. The multi-start procedure worked as follows. The prior expectation for the parameters was perturbed by a small random variate (sampled from a multivariate normal density, with a standard deviation set to a quarter of the corresponding prior). The model was then estimated, and the log evidence (free energy) was recorded. This procedure was repeated eight times, where each time the posteriors of the best model found thus far were used as the priors. Finally, the model with the highest evidence (free energy) was retained. **Supplementary Figure 3** illustrates the effects of this multi-start procedure for a typical subject, for whom all eight model inversions were broadly similar in terms of the Rayleigh parameter (ranging between 17.52 and 18.08). In other words, by initializing the posterior parameter estimates in multiple basins of attraction of multiple local maxima, we could identify the model with the greatest evidence (i.e., variation free energy or marginal likelihood). In this way, we increased the chance of identifying the global maxima. Second, it was necessary to use a particular kind of feature selection that converted discrete symptom scores into continuous variations in amplitude. The requisite smoothing of empirical timeseries used a Gaussian convolution in time (FWHM of eight measurements, using the MATLAB code spm_conv(*data*,8,0)), applied both to the data and model predictions, much in the same way that a link function converts a general linear model into a generalized linear model.

To empirically assess whether the current model setup was overly expressive, using Parametric Empirical Bayesian (PEB) model comparison, we did an automatic search and examined whether any of the parameters within the A and B matrices can be “turned off” at the group level.

#### Demonstrating face validity of parameter estimation

**Figure 3:**
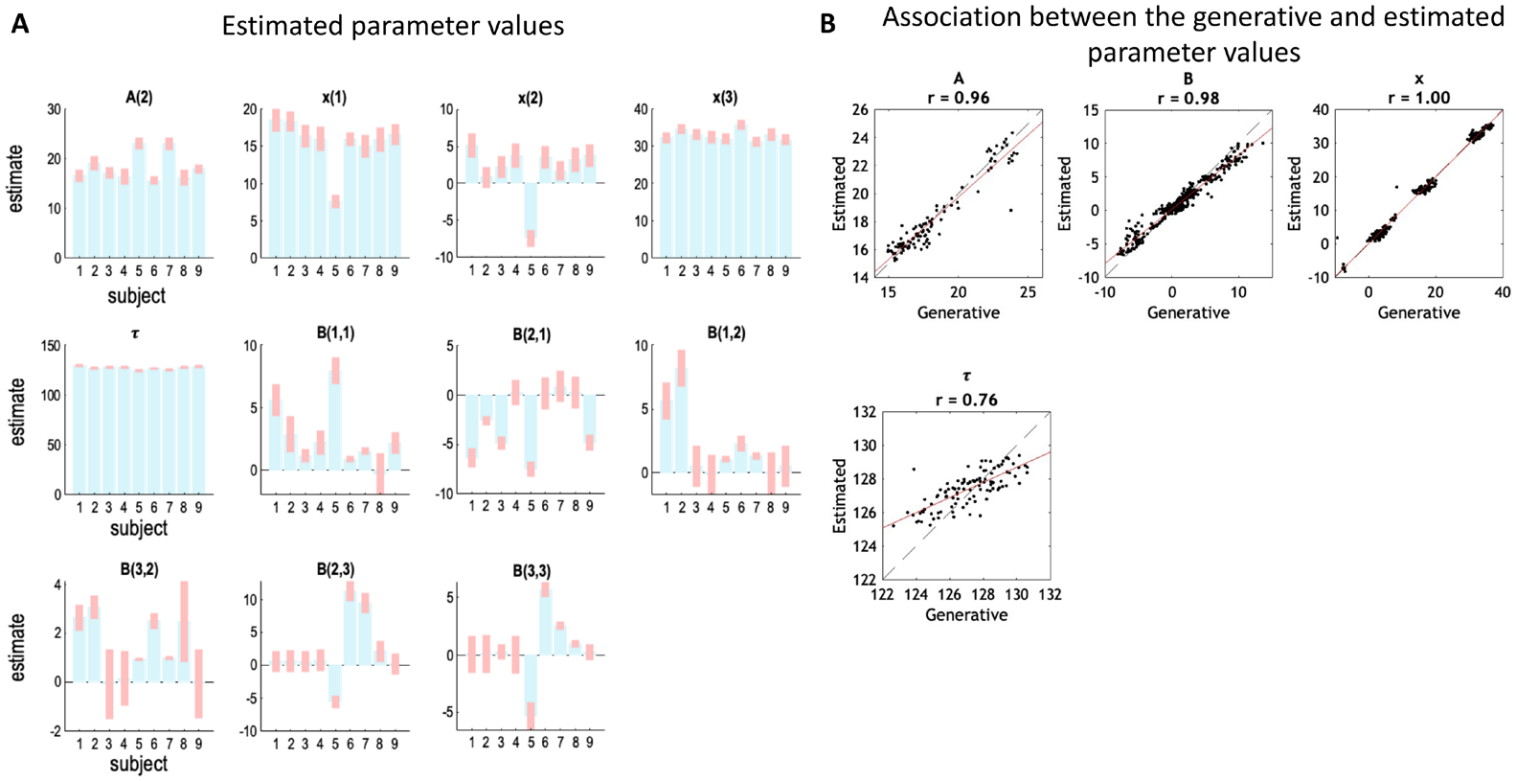
**Subject-level parameter estimation and recovery. A. Estimated parameters.** These bar graphs report the estimated parameters from the model fitting using nine subjects’ empirical data. Blue bars are posterior expected values and error bars are 90% credible intervals (the interval within which the parameter falls with 0.9 probability). **B. Face validity of the parameters.** Parameter values used to generate simulated symptom data were sampled from the posterior densities shown in panel A. This plot shows the estimated parameters from all 108 virtual subjects plotted against the parameters used to generate their data. Each dot is a virtual subject, the dashed line is the diagonal, and the red solid line is the line of best fit. R values denotes Pearson’s correlation coefficients.

Next, we evaluated the face validity of parameter estimation by simulating data using the model and ensuring the parameters used to generate the data could be recovered. The empirical data from the nine human subjects were treated as archetypes of distinct pathology and the ensuring parameters were used to simulate nine groups of virtual subjects (12 subjects per group, n = 9 × 12 = 108 synthetic subjects in total). The parameters of the virtual subjects were sampled from the posterior probability density over each subject’s parameters (as shown in Figure 3A). We then fitted the DCM as above, to estimate new parameters for every virtual subject. To evaluate the face validity of the parameter estimation, we calculated Pearson’s correlation coefficient between the values used to generate the simulated data (true values) and the parameter estimates (i.e., posterior expectations) across the 108 virtual subjects.

#### Demonstrating face validity of group-membership

**Figure 4:**
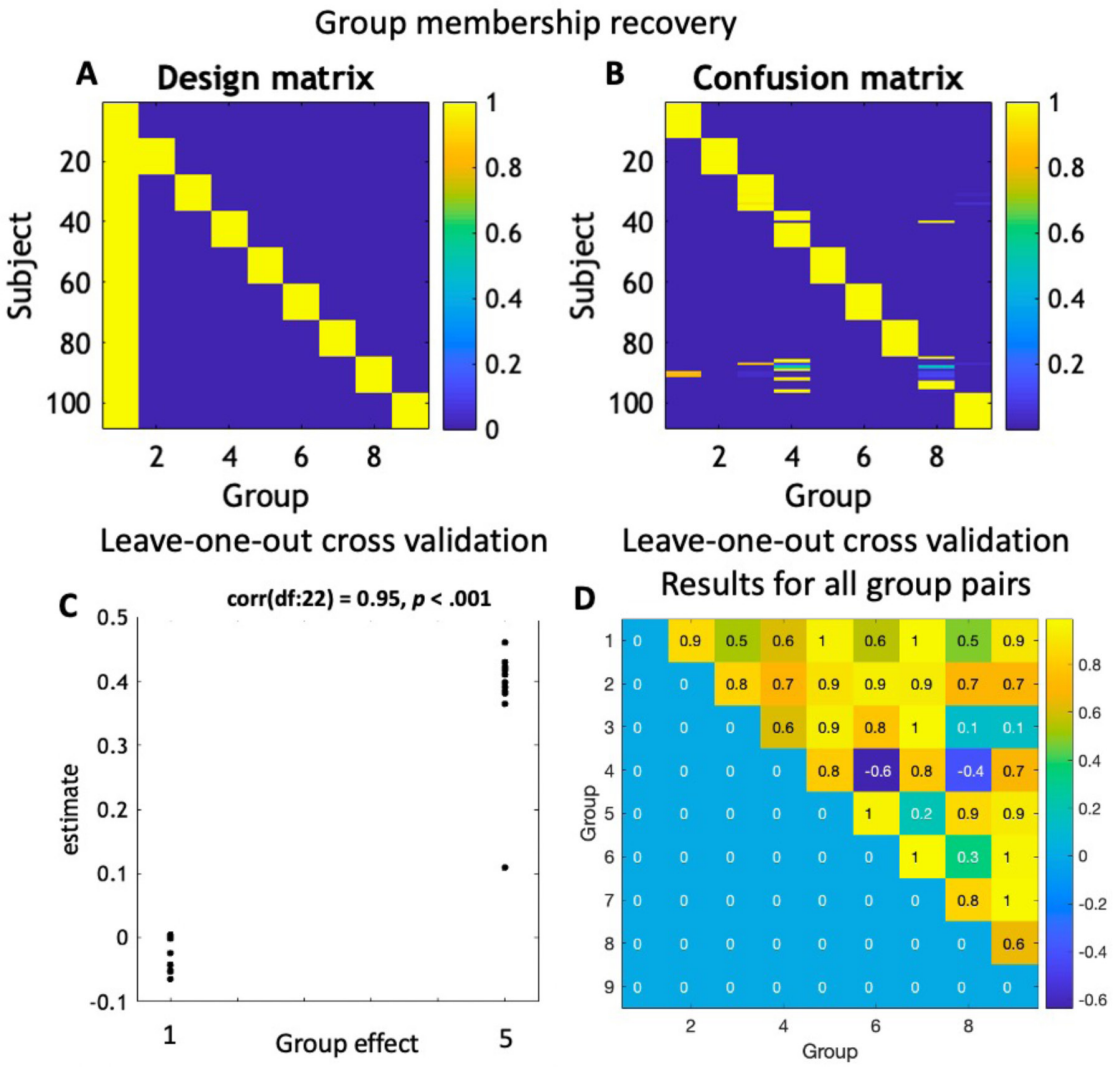
**Assessment of model and group identifiability. A-B. Group membership recovery results.** Assessment of model and group identifiability. **A-B.** Group membership recovery results. Panel A depicts the 108 subjects × 9 groups design matrix (blue = 0, yellow = 1), used in the hierarchical linear regression model (PEB) to test for group membership. Panel B depicts the posterior probability of the estimated group membership ranging from 0 (blue) to 1 (yellow). Each row of this panel is the result of a model comparison, where for each subject, nine PEB models were compared in which the subject was assigned to group 1…9. Coloured lines on the off-diagonal elements indicate mis-classification of subjects. **C.** Illustration of leave-one-out cross-validation, applied to predicting whether subjects belonged to group 1 or group 5. The true group allocation parameter is on the horizontal axis and the estimated group membership parameter is on the vertical axis. **D.** Leave-one-out cross-validation results for all 36 group pairs. The correlation coefficients between the group allocation parameter and the estimated parameter are displayed (black text for values > 0.4).

Finally, we evaluated the accuracy with which we could recover the group membership of each virtual subject using Bayesian model comparison, With parameter estimates from the 108 virtual subjects (from the nine groups) generated above. Using the Parametric Empirical Bayes (PEB) tool in the SPM software package (Zeidman, Jafarian, Corbin, et al., 2019; Zeidman, Jafarian, Seghier, et al., 2019), we specified a general linear model, fitted to the subject-specific parameters of all subjects. In this between-subjects design matrix, the first group was treated as the intercept of the model (i.e., a reference group), and dummy variables (with binary regressors) were included to encode membership in the remaining eight groups (Figure 4A).

We inverted this model and estimated its log evidence, as approximated by the variational free energy. This scores the quality of the model; namely, the log probability of having seen the group-level data, given the between subject or group model. In an empirical setting, a model of this form enables one to test for differences in parameters between subjects, due to diagnosis or clinical measures. Here, with simulated data, we used it to evaluate the confidence with which we could distinguish subjects in different groups. For each virtual subject, we changed the design matrix to assign the subject to group one, group two, etc. We fitted each model to the data and recorded the free energy. Finally, we used Bayes rule (technically, a softmax operator) to convert from the log evidence to the posterior probability for each model. This produced a posterior probability over group membership for each virtual subject, enabling the construction of a *confusion matrix*, whose elements correspond to the probability that a subject from a particular group (rows) would be classified as belonging to each group (columns). Clearly, if the model was identifiable, we would hope to see that the greatest posterior probability for each subject corresponded to the group to which that subject belonged.

#### Leave-one-out cross validation for group membership

The above analyses concern hypothesis testing, i.e., identifying the relative evidence for subjects belonging to one group versus another. Next, we estimated the ability of the model to predict out of sample, by asking how likely is it that a new subject could be correctly classified, i.e., assigned to the correct group. Here, we first used 24 virtual subjects from two arbitrarily chosen groups (12 subjects in each group as mentioned above) and revisited the between-subject analyses above to illustrate its predictive validity: asking whether knowing a new subject’s parameters (specifically, their Rayleigh parameter) would be sufficient to determine which of two groups they were sampled from (e.g., group 1 or group 5). We fitted (i.e., trained) a group-level model, as above, to all virtual subjects except one, who was “left out”. We then predicted the left-out subject’s group allocation (e.g., 0 = group 1, 1 = group 5) on the basis of the likelihood the parameters were sampled from the respective groups. This process was repeated with each subject left out in turn. We then repeat the same procedure for all 36 possible pairs of groups.

#### Data and code availability

The scripts for simulation and model inversion are available in the online Supplementary and upon request.

## Results

### Model fitting to empirical symptom data demonstrates flexibility

Following model inversion, we observed a close correspondence between the predicted symptom scores and the empirical symptom scores for all subjects (Figure 2A & **Supplementary Figure 1**), demonstrating that the DCM is sufficiently expressive to fit a variety of clinical trajectories. These trajectories range from a single episode with an effective recovery, through to fluctuations in symptom expression, over cycles, with an itinerant aspect. The corresponding estimated latent variables are shown in Figure 2B and **Supplementary Figure 2**. Model fitting took 38 to 99 sec for each participant when using a MacBook Air M1 model with 16 GB memory.

The estimated (free) parameters from the above model inversion (Figure 2A) evince some notable features: the Rayleigh parameter, *A*_2_, was largest for subjects 5 and 7, and this was reflected in their fluctuating (cyclical) dynamics (Figure 2B and **Supplementary Figure 2**). The starting values for the pathophysiological states *x* were generally consistent across subjects, except for subject 5 for whom *x*_1_ and *x*_2_ started at a lower value, which could indicate a less symptomatic “disease” trajectory. The time constant *τ* was highly consistent across subjects, whereas the ***B*** parameters that mapped from “pathophysiology” to “psychopathology” varied substantively across subjects. When examining whether any parameters in the ***A*** or ***B*** matrices could be turned off, Bayesian model comparison results showed that one parameter in the B matrix could be safely fixed to 0 (**Supplementary Figure 4**).

### Face validity of subject-level parameter estimation

Figure 3B shows the estimated parameters from all 108 virtual subjects plotted against the parameters used to generate their data. All parameters were recovered with a high degree of accuracy, as reflected by the Pearson’s correlation coefficient (*r* > 0.76). The accuracy was less for the time constant parameter; however, this parameter only varied over a very narrow range. As expected, large generative parameters were pulled back towards their prior expectations, due to the use of Gaussian shrinkage priors.

### Face validity of group-membership

The confusion matrix in Figure 4B shows the inferred group membership for each of the 108 virtual subjects. Almost every subject was assigned to the correct group, demonstrating that they could be distinguished with high confidence. The exceptions were mainly subjects in group eight, the majority of whom were mis-assigned to group four (n = 5), followed by groups one (n = 2) and three (n = 1). One subject in group four was mis-assigned to group eight.

Finally, leave-one-out cross-validation using the 24 virtual subjects from groups 1 and 5 (Figure 4C) showed a highly significant out-of-samples correlation between the estimated group membership and the actual group membership, demonstrating that the effect size was large enough for the Rayleigh parameter to have clinical relevance (in this simulation). Visual inspection revealed that only one out of 24 virtual subject was not correctly classified. Figure 4D shows the out-of-samples correlations for all 36 possible group pairs. Out of the 36, 30 pairs had a moderate or high correlation (correlation coefficient > 0.4).

## Discussion

The time course or trajectory of symptoms contains crucial information about an illness, and hence has been indispensable for nosology research. The current study introduces a nonlinear dynamic (i.e., state space) model that is capable of elucidating the underlying processes that give rise to symptom trajectories. By applying DCM to timeseries of psychotic and mood symptoms—tracked over 48 months following first admission with psychosis—we demonstrated the potential of this approach for a computational nosology. Specifically, we have demonstrated the ability of this hierarchical DCM to explain a variety of clinical trajectories observed in psychotic disorders. Then, using numerical analyses, we demonstrated that a key parameter influencing the cyclicity of latent dynamics can be reliably recovered at the subject-level. Finally, at the group-level, we showed that group membership—based upon specific patterns of parameters—of virtual patients can be recovered. This speaks to the identifiability of the model, given realistic data.

The DCM we employed here is a 3-level generative model. Using a state space model of this sort departs from existing methods for analysis of time course data. Existing approaches either estimate the parameters of static models, such as the intercept and slope of the trajectory, or capture superficial dynamics among data using network models, with no model of the underlying latent pathological states generating those data. In DCM, the dynamics evolve at the level of the underlying disease process, which determine the pattern of symptoms over time. These latent dynamics not only offer a parsimonious account of observed symptoms, but also reveal time-invariant characteristics of latent processes that differ among patients. These characteristics can be used to create a mechanistic nosology (i.e., classify patients according to deep characteristics of their course), forecasting of illness course and response to medications, as well as targets for etiologic research. The DCM approach therefore provides a natural scaffold that can be used to investigate biophysiological and psychological factors whose interactions lead to the manifested symptom trajectories. The 3-level DCM provides a fairly expressive model that allows one to infer latent processes that govern illness time course, which in turn can be targets for psychophysiology research. As noted earlier, when describing the current 3-level DCM, we tentatively used “pathophysiology” and “psychopathology”, following the previous theoretical work (Friston et al., 2017). We envisage that supplementing DCM with likelihood models for other kinds of data—e.g., neurophysiological, pharmacological and psychophysical—may enable one to fine grain and establish the nature of these pathophysiological and psychopathological latent states.

Inspecting the modelled latent variables can provide insights into how each subject’s model explained their data. For instance, in Figure 2, we can see that subject s1 had both psychosis and depression which resolved at the same time, early in the study period. This was explained by the model in terms of an exponential reduction in their psychosis and depression psychopathology latent variables, in the absence of exogenous inputs. By contrast, subject s9 had only psychosis, which persisted longer before resolving. The model explained this symptom trajectory differently, in terms of an exogenous input (i.e., a life event), which drove the psychopathology latent variable. Why was exogenous input estimated to be important for one subject but not the other? We can answer this from several perspectives. First, when fitting each model, parameter values are chosen that maximize the free energy. The free energy can be decomposed into the accuracy of the model minus its complexity, where complexity is how far the parameters have diverged from their priors (the KL-divergence). Thus, after equating for accuracy, solutions will be chosen that are as simple as possible, using the fewest effective number of parameters. From this perspective, whereas s1’s symptoms could be explained in terms of regular fluctuations in psychopathology states alone, this was insufficient to explain s9’s symptoms, and the added involvement of exogenous inputs was required. In other words, the added complexity cost of invoking exogenous effects was offset by an increase in accuracy. Second, during model fitting, there are likely to be local maxima in the landscape of parameters, which can cause different subjects to end up in different local maxima. To mitigate this, we deployed a multiple-starting scheme, where eight different starting sets of parameters were specified per subject, and the best solution was retained. Nevertheless, subjects s9 and s1 may still have fallen into distinct local maxima, favouring solutions with and without exogenous inputs respectively. A standard way to address this would be to estimate every subject’s parameters (using a multi-start scheme if desired), then re-estimate all subjects using the group average parameters as a starting point (Friston et al., 2015; Litvak et al., 2015). This generally ensures all subjects converge to the same local optimum, providing more consistent solutions (this is implemented in the SPM MATLAB function spm_dcm_peb_fit). Third, the model’s propensity to explain effects in terms of endogenous dynamics relative to exogenous inputs could be optimised, by adjusting appropriate prior precisions. For example, a higher prior precision of a parameter increases the complexity cost of that parameter deviating from its prior expectation. Prior precisions would then be optimised under (usually uninformative) hyperpriors.

Effects of specific medications, stressors, and triggers can be studied directly within the DCM framework. These factors can be modeled as known exogenous inputs, which goes beyond the scope of the present study. Modeling known inputs could identify symptom trajectories that respond to one drug versus another, and thus be used to forecast treatment response based on symptom history of a patient. Also, DCM can clarify how these factors modulate the illness (Friston et al., 2017). For example, it could be used to ask whether dopamine D2 antagonists increase the threshold at which psychotic symptoms manifest or whether they rather blunt fluctuations in psychotic symptoms, as both of these mechanisms can produce symptom remission. A nice example of this kind of application of DCM can be found in the study of epilepsy, where the temporal evolution of seizures has been captured as trajectories through a space of neural model parameters (Rosch et al., 2018).

In the current example, we allowed a subset of the parameters in the DCM to vary over subjects, focusing on the Rayleigh parameter, given its key role in underwriting the cyclicity of trajectories, which is of high importance in clinical research. Varying this parameter can result in drastically different cyclicity patterns. For future studies, one could consider combinations of free parameters, depending on the nature of the data and the research questions. For instance, instead of focusing on the Rayleigh parameter in ***A***, researchers may allow the parameters in the ***B*** matrix to vary across individuals. Such a choice could be used to test individual differences in the mapping from psychophysiology to psychopathology, given the same pathophysiology states. Crucially, the choice of whether to allow a parameter to vary or not is guided by model comparison and is therefore a question of empirical study. More specifically, one would compare models in which a certain parameter — or combination of parameters — were free to be informed by the data, against models in which they were fixed to default values. If the model evidence increases when allowing a parameter to vary, then this is evidence that the parameter in question plays a role in explaining symptom expression. If the model evidence decreases, this suggests the parameter contributes only to the complexity of the model, without increasing its accuracy. In other words, Bayesian model comparison can be used to explore different models defined in terms of which parameters are fixed and which parameters are free; automatically fixing or removing redundant parameters. In the current study, we illustrated how using automatic searching implemented in PEB has identified that one of the parameters in the ***B*** matrix can be safely fixed to zero in the sample of the 9 subjects. Clearly, this depends upon the nature and quality of the data at hand, i.e., for some data simpler models may be optimal and more expressive models may be required for other data.

As mentioned above, a particularly interesting factor to examine, in future research, is the exogeneous input. In the current illustrations, this exogenous input was specified as a general linear model with a design matrix ***D*** comprising a discrete cosine transform basis set. Partly constrained by our data, the exogenous input was then inferred from the data. Future studies—with a detailed assessment of these external events such as interventions and life stressors— would enable the exogeneous inputs to be treated as a known variable. Practically, this involved specifying priors over the exogenous input, but can be specified with greater or lesser precision. Under such circumstances, one can use the current 3-level DCM to investigate how pathophysiology and/or psychopathology would vary as a function of known exogeneous inputs and the associated nosology mechanisms.

There are two points worth considering when validating the current model in future research. The approach we took to simulation was to generate data from 108 virtual subjects, whose parameters were sampled from one of nine virtual groups. We then fitted the model to these simulated data, and confirmed accurate recovery of parameters and—most importantly for testing hypotheses—accurate discrimination of models using Bayesian model comparison. In following this procedure, there are challenges of assessing “face validity”. First, counter-intuitively, there may be a set of parameters that better explains the data (i.e., with lower complexity) than the parameters that were actually used to generate those data, because they afford a better log-evidence. This would appear as a failure of face validation, which could lead one to under-estimate the performance of the model (Litvak et al., 2019). A second challenge regards parameter selection for simulating data. To validate the estimation of the posterior density (i.e., credible intervals), one may wish to use a large number of “ground truths” for data simulation, which are sampled from the prior density. This has been referred to as Simulation-Based Calibration (Gelman et al., 2020; Talts et al., 2018). This has the advantage of being comprehensive, while having the disadvantage that unrealistic data may be generated when relatively uninformative priors are used, as was the case here. Hence, we opted to use empirical posteriors as the basis for our simulations, as they ensure a level of ecologically validity.

The approach taken here may be contrasted against state-of-the-art machine learning methods, such as variational auto-encoders or deep/recurrent neural networks. Here, our intention was to provide tools for scoring the evidence for different hypotheses, where each hypothesis is expressed as a *generative model* of the data. A generative model comprises a likelihood function and a set of priors, which are prerequisites for calculating the evidence. For this reason, the models here are specified according to the experimenter’s hypotheses, with flexibility where certain processes are unknown (e.g., by inferring unknown exogenous inputs using a DCT basis set). For this reason, dynamic causal models are readily explainable with a clear—if coarse-grained—interpretation of their parameters. By contrast, the priority for machine learning methods tends to be to infer the latent structure of some data in order to classify or make predictions; rather than score the model evidence or provide explainable parameters. Nevertheless, there are commonalities across our approach and deep neural networks. Both seek to infer latent variables that explain the data, which are organised hierarchically with increasing levels of abstraction. Similar variational Bayes methods as used in DCM form the basis of the variational auto-encoder, where the data are mapped to the parameters of a latent probability distribution. Finally, a potentially interesting future direction for the model presented here, which is a point of contact between approaches, would be to apply amortized inference. This is where a neural network is applied to map between the parameters in the DCM and the observed data. This could enable integration of new patients’ data without having to re-estimate the full model each time.

We have appealed to the explainability afforded by generative modelling with DCM. This ‘explainability’ calls for some qualification. We use explainability in the general sense that we can explain the data in terms of some latent or hidden causes (i.e., states and parameters of a generative model). However, this does not mean that the latent states and parameters can be directly mapped onto biophysical quantities. Biophysical explainability requires the model to be parameterised in a biologically plausible way—so that one can clearly identify the mapping from model parameters to rate constants, connection strengths and other neurobiological constructs. In general, this means there is a distinction between biophysical and phenomenological DCMs (Pereira et al., 2021), which depends upon the degree of abstraction or course graining of the latent states. The current DCM is a phenomenological DCM because it does not commit to particular biophysical mechanisms. Rather, it provides the opportunity to summarise a potentially complex timeseries in terms of a small number of time-invariant parameters, and thereby offers a parsimonious model. The idea then is to associate these parameters with independent measures of pathophysiology and psychopathology by examining their correlations over subjects. This use of the estimates of DCM parameters to identify the characteristic differences — that speak to underlying mechanisms – has been referred to as generative embedding (Brodersen et al., 2014). An objective of future research using DCM in the current context is to establish its construct validity in relation to schemes such as the Hierarchical Taxonomy of Psychopathology (Kotov, Krueger, et al., 2017) and, ultimately, the underlying neurobiology.

Another point for consideration for future research is that when implementing our approach, it may be important to evaluate the robustness of the model to the kinds of sampling noise typically encountered, and to consider incorporating any such random effects into the model. For instance, if patients mis-reported their symptoms, this would change the probabilistic mapping from latent psychopathology to symptom scores. In the current model, this mapping is captured by a sigmoid function, *s_i_(t*), whose parameters could be treated as free parameters and optimised, as needed, to accommodate random effects of this sort.

The current study has several limitations. First, the DCM setup in the current study may not be apt for all forms of psychopathology. Rather, the current DCM is best regarded as a framework for psychiatric hypothesis testing, as described above. Second, people unfamiliar with dynamic causal modelling may be asking how one validates the implicit variational procedures and the ensuing estimators. In brief, the main usage of dynamic causal modelling is to compare the evidence for competing models or hypotheses about how data were caused. This means one has to evaluate the model evidence or marginal likelihood of a model, which, in general, is an intractable problem. This is why the variational free energy (a.k.a., evidence lower bound) was introduced, to convert an impossible marginalisation problem into a tractable optimisation problem (Beal, 2003; Feynman, 2018). The alternative to variational Bayes is to relax assumptions about the functional form of probability distributions and use sampling; such as in Markov Chain Monte Carlo (MCMC) procedures or Approximate Bayesian Computation: e.g., (Rubin, 1984). These sampling procedures provide posterior distributions over model parameters. However, they do not resolve the intractable problem of evaluating model evidence. Although approximations such as the Bayesian and Akaike Information Criteria (and their variants) can be used in the context of sampling (Spiegelhalter et al., 2002), they are notoriously poor approximations to model evidence; especially in relation to variational free energy (Penny, 2012). Effectively, this means that there is no alternative to the variational approach taken by dynamic causal modelling, if the agenda is to compare models. Having said this, sampling procedures can be useful to confirm the form of the posterior distributions used in dynamic causal modelling: see for example (Chumbley et al., 2007; Penny & Sengupta, 2016; Sengupta et al., 2016). Third, for demonstration purposes, only a small number of subjects were considered in this report. Subsequent papers will apply the methods described in the current report to a larger sample size. These application papers will establish empirical priors over the model parameters and indeed, the form of the model. In short, this paper can be read as a technical foundation for subsequent applications. Empirical results should not be generalized beyond the illustrative analyses included here. Forth, we had access to 48 months of illness course that was carefully tracked in a longitudinal study, which is difficult to collect. Fortunately, electronic health records, mobile monitoring, and experience sampling technologies make it feasible to gather time course data needed for the DCM.

In conclusion, the current study considers the first step in applying DCM, characterized by multi-level, dynamic, and nonlinear models, to psychiatric nosology. With both empirical data and numerical analyses, we showed that the approach has the potential to elucidate a more mechanistic nosology than current heuristic diagnoses. The resulting quantitative classification promises to facilitate etiology and pathophysiology research as well as improve the forecasting of illness course and treatment response. More broadly, there is a prescient opportunity to apply dynamic modeling in psychopathology research, and the current study introduces a set of tools that may prove useful in this regard.

## Additional file

The additional file for this article can be found as follows:

10.5334/cpsy.94.s1 Supplementary Methods and Materials. Data and code.

## References

[1] Angst, J., Gamma, A., Benazzi, F., Ajdacic, V., Eich, D., & Rössler, W. (2003). Toward a re-definition of subthreshold bipolarity: epidemiology and proposed criteria for bipolar-II, minor bipolar disorders and hypomania. Journal of affective disorders, 73(1–2), 133–146. DOI: 10.1016/S0165-0327(02)00322-1 12507746

[2] Asparouhov, T., Hamaker, E. L., & Muthén, B. (2018). Dynamic structural equation models. Structural Equation Modeling: A Multidisciplinary Journal, 25(3), 359–388. DOI: 10.1080/10705511.2017.1406803

[3] Beal, M. J. (2003). Variational algorithms for approximate Bayesian inference. University of London, University College London (United Kingdom).

[4] Beltz, A. M., & Gates, K. M. (2017). Network mapping with GIMME. Multivariate behavioral research, 52(6), 789–804. DOI: 10.1080/00273171.2017.1373014 29161187 PMC6181449

[5] Brodersen, K. H., Deserno, L., Schlagenhauf, F., Lin, Z., Penny, W. D., Buhmann, J. M., & Stephan, K. E. (2014). Dissecting psychiatric spectrum disorders by generative embedding. Neuroimage Clin, 4, 98–111. DOI: 10.1016/j.nicl.2013.11.002 24363992 PMC3863808

[6] Bromet, E. J., Schwartz, J. E., Fennig, S., Geller, L., Jandorf, L., Kovasznay, B., Lavelle, J., Miller, A., Pato, C., Ram, R., & Rich, C. (1992). The Epidemiology of Psychosis: The Suffolk County Mental Health Project. Schizophrenia bulletin, 18(2), 243–255. DOI: 10.1093/schbul/18.2.243 1621071

[7] Chumbley, J. R., Friston, K. J., Fearn, T., & Kiebel, S. J. (2007). A Metropolis–Hastings algorithm for dynamic causal models. Neuroimage, 38(3), 478–487. DOI: 10.1016/j.neuroimage.2007.07.028 17884582

[8] Ciompi, L. (1980). Catamnestic long-term study on the course of life and aging of schizophrenics. Schizophrenia bulletin, 6(4), 606–618. DOI: 10.1093/schbul/6.4.606 7444392

[9] Cuthbert, B. N., & Insel, T. R. (2013). Toward the future of psychiatric diagnosis: the seven pillars of RDoC. BMC medicine, 11(1), 1–8. DOI: 10.1186/1741-7015-11-126 23672542 PMC3653747

[10] Daunizeau, J., David, O., & Stephan, K. E. (2011). Dynamic causal modelling: a critical review of the biophysical and statistical foundations. Neuroimage, 58(2), 312–322. DOI: 10.1016/j.neuroimage.2009.11.062 19961941

[11] Duncan, T. E., Duncan, S. C., & Strycker, L. A. (2011). An introduction to latent variable growth curve modeling: concepts, issues, and applications (Second edition). Psychology Press. DOI: 10.4324/9780203879962

[12] Epskamp, S., Waldorp, L. J., Mottus, R., & Borsboom, D. (2018). The Gaussian Graphical Model in Cross-Sectional and Time-Series Data. Multivariate Behav Res, 53(4), 453–480. DOI: 10.1080/00273171.2018.1454823 29658809

[13] Feynman, R. P. (2018). Statistical mechanics: a set of lectures. CRC press. DOI: 10.1201/9780429493034

[14] First, M. B., Rebello, T. J., Keeley, J. W., Bhargava, R., Dai, Y., Kulygina, M., Matsumoto, C., Robles, R., Stona, A. C., & Reed, G. M. (2018). Do mental health professionals use diagnostic classifications the way we think they do? A global survey. World Psychiatry, 17(2), 187–195. https://www.ncbi.nlm.nih.gov/pmc/articles/PMC5980454/pdf/WPS-17-187.pdf. DOI: 10.1002/wps.20525 29856559 PMC5980454

[15] Fisher, A. J., Reeves, J. W., Lawyer, G., Medaglia, J. D., & Rubel, J. A. (2017). Exploring the Idiographic Dynamics of Mood and Anxiety via Network Analysis. Journal of abnormal psychology (1965), 126(8), 1044–1056. DOI: 10.1037/abn0000311 29154565

[16] Frässle, S., Harrison, S. J., Heinzle, J., Clementz, B. A., Tamminga, C. A., Sweeney, J. A., Gershon, E. S., Keshavan, M. S., Pearlson, G. D., & Powers, A. (2021). Regression dynamic causal modeling for resting-state fMRI. Human brain mapping, 42(7), 2159–2180. DOI: 10.1002/hbm.25357 33539625 PMC8046067

[17] Friston, K., Costello, A., & Pillay, D. (2020). ‘Dark matter’, second waves and epidemiological modelling. BMJ Glob Health, 5(12), e003978. DOI: 10.1136/bmjgh-2020-003978 PMC7745338 33328201

[18] Friston, K., Mattout, J., Trujillo-Barreto, N., Ashburner, J., & Penny, W. (2007). Variational free energy and the Laplace approximation. Neuroimage, 34(1), 220–234. DOI: 10.1016/j.neuroimage.2006.08.035 17055746

[19] Friston, K., Zeidman, P., & Litvak, V. (2015). Empirical Bayes for DCM: A Group Inversion Scheme. Front Syst Neurosci, 9, 164. DOI: 10.3389/fnsys.2015.00164 26640432 PMC4661273

[20] Friston, K. J., Harrison, L., & Penny, W. (2003). Dynamic causal modelling. Neuroimage, 19(4), 1273–1302. DOI: 10.1016/S1053-8119(03)00202-7 12948688

[21] Friston, K. J., Parr, T., Zeidman, P., Razi, A., Flandin, G., Daunizeau, J., Hulme, O. J., Billig, A. J., Litvak, V., Moran, R. J., Price, C. J., & Lambert, C. (2020). Dynamic causal modelling of COVID-19. Wellcome Open Research, 5(89), 89. DOI: 10.12688/wellcomeopenres.15881.1 32832701 PMC7431977

[22] Friston, K. J., Redish, A. D., & Gordon, J. A. (2017). Computational Nosology and Precision Psychiatry. Computational psychiatry, 1, 2–23. DOI: 10.1162/cpsy_a_00001 29400354 PMC5774181

[23] Gandolfi, D., Pagnoni, G., Filippini, T., Goffi, A., Vinceti, M., D’Angelo, E., & Mapelli, J. (2021). Modeling Early Phases of COVID-19 Pandemic in Northern Italy and Its Implication for Outbreak Diffusion [Original Research]. Frontiers in Public Health, 9(1946). DOI: 10.3389/fpubh.2021.724362 PMC8716563 34976909

[24] Gelman, A., Vehtari, A., Simpson, D., Margossian, C. C., Carpenter, B., Yao, Y., Kennedy, L., Gabry, J., Bürkner, P.-C., & Modrák, M. (2020). Bayesian workflow. arXiv preprint arXiv:2011.01808.

[25] Gordon, J. A., & Redish, A. D. (2016). On the cusp. Current challenges and promises in psychiatry. In A. D., Redish & J. A. Gordon (Eds.), Computational psychiatry: New perspectives on mental illness (pp. 3–14). Cambridge, MA: MIT Press. DOI: 10.7551/mitpress/9780262035422.003.0001

[26] Hyman, S. E. (2010). The diagnosis of mental disorders: the problem of reification. Annual review of clinical psychology, 6, 155–179. DOI: 10.1146/annurev.clinpsy.3.022806.091532 17716032

[27] Kotov, R., Fochtmann, L., Li, K., Tanenberg-Karant, M., Constantino, E. A., Rubinstein, J., Perlman, G., Velthorst, E., Fett, A.-K. J., & Carlson, G. (2017). Declining clinical course of psychotic disorders over the two decades following first hospitalization: evidence from the Suffolk County Mental Health Project. American Journal of Psychiatry, 174(11), 1064–1074. DOI: 10.1176/appi.ajp.2017.16101191 28774193 PMC5767161

[28] Kotov, R., Jonas, K. G., Lian, W., Docherty, A. R., & Carpenter, W. T. (2022). Reconceptualizing schizophrenia in the Hierarchical Taxonomy Of Psychopathology (HiTOP). Schizophr Res, 242, 73–77. DOI: 10.1016/j.schres.2022.01.053 35144862 PMC9675950

[29] Kotov, R., Krueger, R. F., Watson, D., Achenbach, T. M., Althoff, R. R., Bagby, R. M., Brown, T. A., Carpenter, W. T., Caspi, A., & Clark, L. A. (2017). The Hierarchical Taxonomy of Psychopathology (HiTOP): a dimensional alternative to traditional nosologies. Journal of abnormal psychology, 126(4), 454. DOI: 10.1037/abn0000258 28333488

[30] Kotov, R., Leong, S. H., Mojtabai, R., Erlanger, A. C. E., Fochtmann, L. J., Constantino, E., Carlson, G. A., & Bromet, E. J. (2013). Boundaries of Schizoaffective Disorder: Revisiting Kraepelin. JAMA psychiatry (Chicago, Ill.), 70(12), 1276–1286. DOI: 10.1001/jamapsychiatry.2013.2350 24089086

[31] Krueger, R. F., Kotov, R., Watson, D., Forbes, M. K., Eaton, N. R., Ruggero, C. J., Simms, L. J., Widiger, T. A., Achenbach, T. M., & Bach, B. (2018). Progress in achieving quantitative classification of psychopathology. World Psychiatry, 17(3), 282–293. DOI: 10.1002/wps.20566 30229571 PMC6172695

[32] Krystal, J. H., Murray, J. D., Chekroud, A. M., Corlett, P. R., Yang, G., Wang, X.-J., & Anticevic, A. (2017). Computational psychiatry and the challenge of schizophrenia. Schizophrenia bulletin, 43(3), 473–475. DOI: 10.1093/schbul/sbx025 28338845 PMC5464204

[33] Lane, S. T., Gates, K. M., Pike, H. K., Beltz, A. M., & Wright, A. G. C. (2019). Uncovering General, Shared, and Unique Temporal Patterns in Ambulatory Assessment Data. Psychological methods, 24(1), 54–69. DOI: 10.1037/met0000192 30124300 PMC6433550

[34] Litvak, Y., Biess, A., & Bar-Hillel, A. (2019). Learning pose estimation for high-precision robotic assembly using simulated depth images. 2019 International Conference on Robotics and Automation (ICRA). DOI: 10.1109/ICRA.2019.8794226

[35] Litvak, V., Garrido, M., Zeidman, P., & Friston, K. (2015). Empirical Bayes for Group (DCM) Studies: A Reproducibility Study. Front Hum Neurosci, 9, 670. DOI: 10.3389/fnhum.2015.00670 26733846 PMC4686807

[36] Nagin, D. S., & Odgers, C. L. (2010). Group-based trajectory modeling in clinical research. Annual review of clinical psychology, 6(1), 109–138. DOI: 10.1146/annurev.clinpsy.121208.131413 20192788

[37] Nelson, B., McGorry, P. D., Wichers, M., Wigman, J. T. W., & Hartmann, J. A. (2017). Moving From Static to Dynamic Models of the Onset of Mental Disorder: A Review. JAMA psychiatry, 74(5), 528–534. DOI: 10.1001/jamapsychiatry.2017.0001 28355471

[38] Papadopoulou, M., Cooray, G., Rosch, R., Moran, R., Marinazzo, D., & Friston, K. (2017). Dynamic causal modelling of seizure activity in a rat model. Neuroimage, 146, 518–532. DOI: 10.1016/j.neuroimage.2016.08.062 27639356

[39] Penny, W., & Sengupta, B. (2016). Annealed importance sampling for neural mass models. PLOS Computational Biology, 12(3), e1004797. DOI: 10.1371/journal.pcbi.1004797 26942606 PMC4778905

[40] Penny, W. D. (2012). Comparing dynamic causal models using AIC, BIC and free energy. Neuroimage, 59(1), 319–330. DOI: 10.1016/j.neuroimage.2011.07.039 21864690 PMC3200437

[41] Pereira, I., Frassle, S., Heinzle, J., Schobi, D., Do, C. T., Gruber, M., & Stephan, K. E. (2021). Conductance-based dynamic causal modeling: A mathematical review of its application to cross-power spectral densities. Neuroimage, 245, 118662. DOI: 10.1016/j.neuroimage.2021.118662 34687862

[42] Rosch, R. E., Hunter, P. R., Baldeweg, T., Friston, K. J., & Meyer, M. P. (2018). Calcium imaging and dynamic causal modelling reveal brain-wide changes in effective connectivity and synaptic dynamics during epileptic seizures. PLoS Comput Biol, 14(8), e1006375. DOI: 10.1371/journal.pcbi.1006375 30138336 PMC6124808

[43] Rubin, D. B. (1984). Bayesianly justifiable and relevant frequency calculations for the applied statistician. The Annals of Statistics, 1151–1172. DOI: 10.1214/aos/1176346785

[44] Sengupta, B., Friston, K. J., & Penny, W. D. (2016). Gradient-based MCMC samplers for dynamic causal modelling. Neuroimage, 125, 1107–1118. DOI: 10.1016/j.neuroimage.2015.07.043 26213349 PMC4692453

[45] Shil’Nikov, A., Shil’Nikov, L., & Turaev, D. (1993). Normal forms and Lorenz attractors. International Journal of Bifurcation and Chaos, 3(05), 1123–1139. DOI: 10.1142/S0218127493000933

[46] Spiegelhalter, D. J., Best, N. G., Carlin, B. P., & Van Der Linde, A. (2002). Bayesian measures of model complexity and fit. Journal of the royal statistical society: Series b (statistical methodology), 64(4), 583–639. DOI: 10.1111/1467-9868.00353

[47] Talts, S., Betancourt, M., Simpson, D., Vehtari, A., & Gelman, A. (2018). Validating Bayesian inference algorithms with simulation-based calibration. arXiv preprint arXiv:1804.06788.

[48] Taylor, D. (2016). Prescribing according to diagnosis: how psychiatry is different. World Psychiatry, 15(3), 224. DOI: 10.1002/wps.20343 27717274 PMC5032505

[49] Wang, X.-J., & Krystal, J. H. (2014). Computational Psychiatry. Neuron (Cambridge, Mass.), 84(3), 638–654. DOI: 10.1016/j.neuron.2014.10.018 PMC4255477 25442941

[50] Waszczuk, M. A., Zimmerman, M., Ruggero, C., Li, K., MacNamara, A., Weinberg, A., Hajcak, G., Watson, D., & Kotov, R. (2017). What do clinicians treat: Diagnoses or symptoms? The incremental validity of a symptom-based, dimensional characterization of emotional disorders in predicting medication prescription patterns. Comprehensive Psychiatry, 79, 80–88. DOI: 10.1016/j.comppsych.2017.04.004 28495012 PMC5643213

[51] Wright, A. G., & Woods, W. C. (2020). Personalized models of psychopathology. Annual review of clinical psychology, 16, 49–74. DOI: 10.1146/annurev-clinpsy-102419-125032 32070120

[52] Zeidman, P., Friston, K., & Parr, T. (2022). A primer on Variational Laplace. DOI: 10.31219/osf.io/28vwh PMC10951963 37544417

[53] Zeidman, P., Jafarian, A., Corbin, N., Seghier, M. L., Razi, A., Price, C. J., & Friston, K. J. (2019). A guide to group effective connectivity analysis, part 1: first level analysis with DCM for fMRI. Neuroimage, 200, 174–190. DOI: 10.1016/j.neuroimage.2019.06.031 31226497 PMC6711459

[54] Zeidman, P., Jafarian, A., Seghier, M. L., Litvak, V., Cagnan, H., Price, C. J., & Friston, K. J. (2019). A guide to group effective connectivity analysis, part 2: Second level analysis with PEB. Neuroimage, 200, 12–25. DOI: 10.1016/j.neuroimage.2019.06.032 31226492 PMC6711451

