# Supplementary material for: Inferring Trajectories of Psychotic Disorders Using Dynamic Causal Modeling
Source: Comput Psychiatr. 2023 Aug 28;7(1):60–75. doi: 10.5334/cpsy.94 (PMC11104383; doi:10.5334/cpsy.94)
Supplement: Supplementary Methods and Materials. — Data and code. [file cpsy-7-1-94-s1.zip › cpsy-94_jin-s1/data/data.docx]

**Supplementary Methods and Materials**

*Notes about the generative model.*

The generative model consists of 4 sets of time-variant variables ((observable) symptoms, (latent) psychopathology, (latent) pathophysiology, and the (observable) number of assessments) and 6 sets of time-invariant parameters (***A, B, C, x, u, t***). See **Table 1** for the glossary. In the current study, the exogeneous inputs were an estimated timeseries vector. But the exogeneous inputs are not latent variable. We remained agnostic regarding the exact nature of this vector, but this can be a summary of life events, such as life stressors, psychological and pharmacological interventions etc.

**Supplementary Figures**


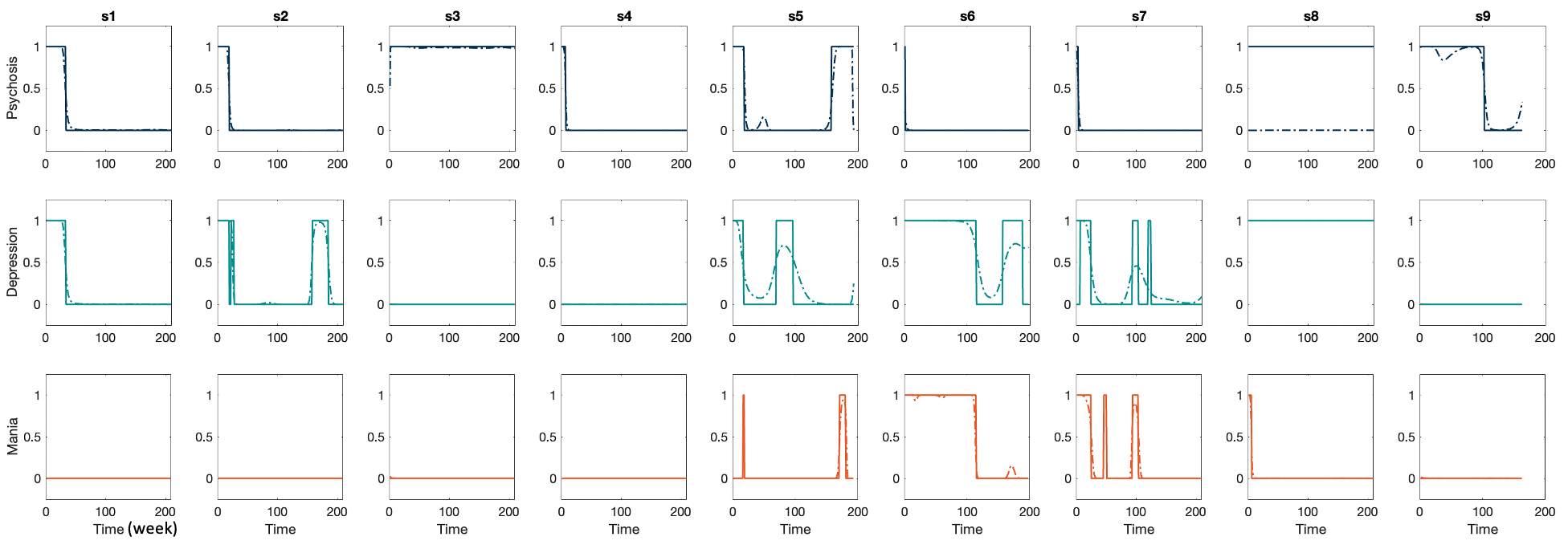


**SFigure 1. Actual and estimated symptoms for all subjects.** Solid lines depict actual symptom data, and dash lines depict estimated symptom data.


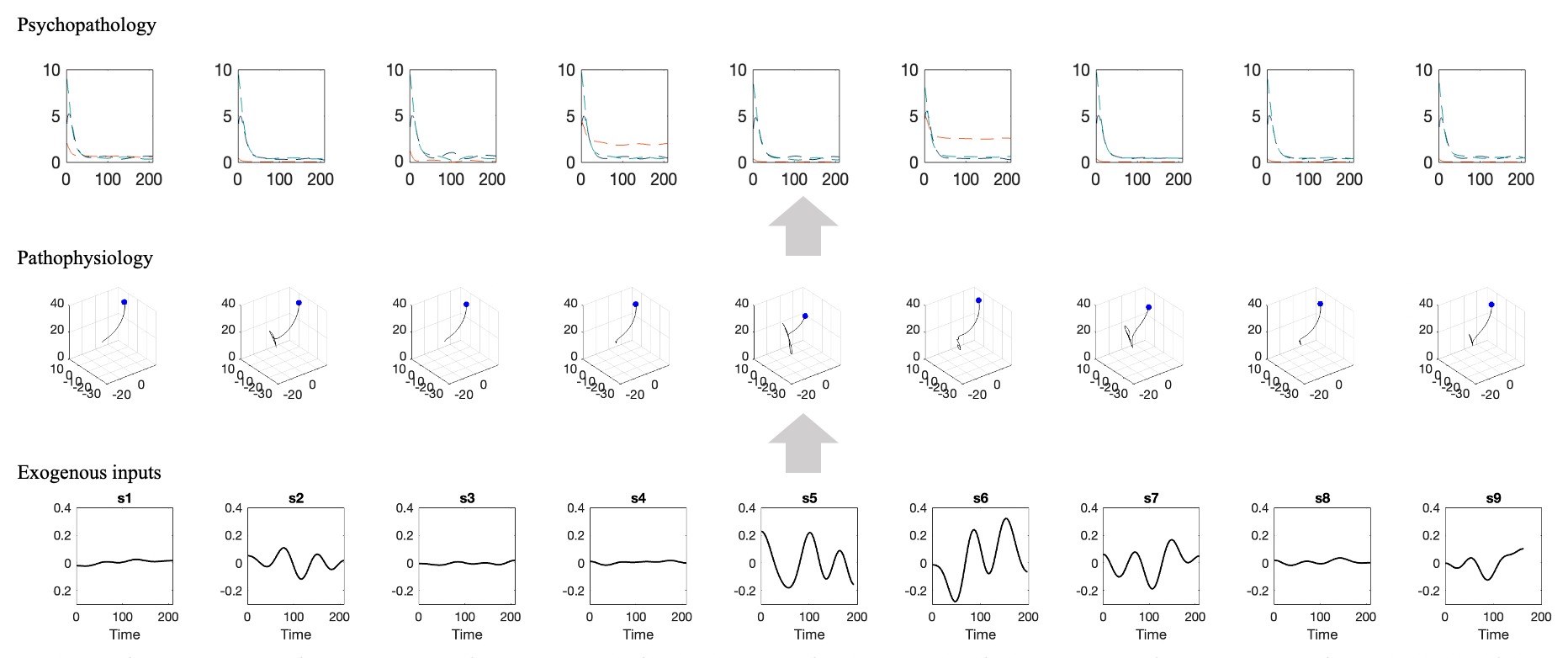


**SFigure 2. Estimated latent variables and exogeneous inputs for all subjects.** From left to right shows subject 1 to subject 9.


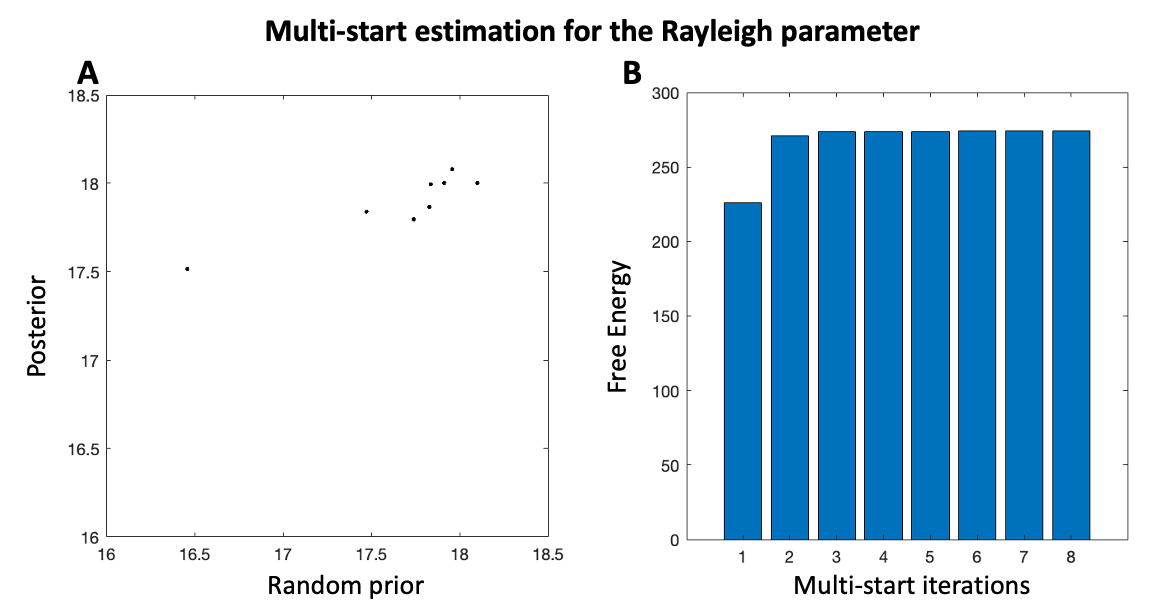


**SFigure 3.** Performance of the multi-start model inversion. **A.** The prior and posterior expected values for the 8 iterations. **B.** Their corresponding free energy.


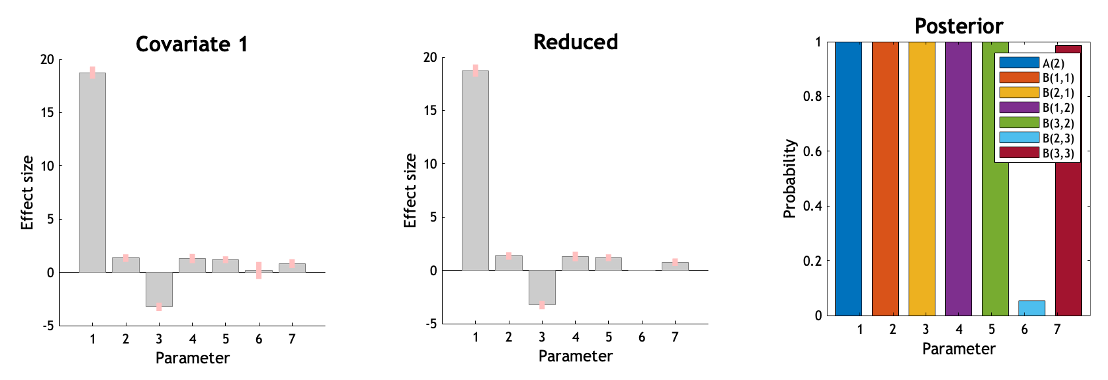


**SFigure 4.** Model reduction using PEB. The left panel shows estimated values of seven free parameters (labelled in the legend of the right panel) following Bayesian model reduction. Pink bars are 90% credible intervals. This shows that the sixth parameter, ***B***(2,3), was fixed to its prior expectation (zero) without reducing the free energy at the group level. The right panel shows the corresponding posterior probability for each parameter being non-zero, confirming strong evidence for every parameter except for ***B***(2,3).

**Matlab code for the structure of the generative model**

% Set up the generative model

%==========================================================================

% dynamics and parameters of a Lorentz system (with Jacobian)

%==========================================================================

% dxdt = f(x,v) Underlying dynamics with exogenous inputs

% s = h(d(x)) + e Observer model generating observed data

%--------------------------------------------------------------------------

% level 1: Thresholding function (M.h) that converts continuous states of

% psychopathology into discrete scores (here between 0 and 1) with

% thresholds parameterised by P.u. This function can be changed to apply

% multiple thresholds to generate discrete scales of the sort found in

% clinical instruments.

%--------------------------------------------------------------------------

M.h = @(x,P)spm_phi([x(:,1) - P.u(1), x(:,2) - P.u(2), x(:,3) - P.u(3)]*8);

% level 2: the level that generates latent (psychopathology)causes y = g(x)

% from(pathophysiological) states (x) that are subject to interventions (v).

% The first equation (M.f) models the dynamics or flow of underlying

% pathophysiology using a Lorentz system, while the second equation (M.d)

% extracts a subset of physiological states and mixes them to generate the

% psychological states (y). The parameters of these functions determine the

% underlying dynamics of the disease (P.A) and its psychopathological

% manifestation (P.B)

%--------------------------------------------------------------------------

M.f = @(x,v,P,M)[-P.A(1) P.A(1) 0; ((1 - v*P.A(3))*P.A(2) - x(3)) -1 0; x(2) 0 -8/3]*x/P.t;

M.d = @(x,P) x*P.B/16;

% level 3: the level that perturbed the physiological dynamics by

% intervening on the parameters governing flow. These interventions or

% exogenous inputs could be regarded as life events or pharmacological

% interventions. This exogenous input variable (M.v) is smooth and

% fluctuating changes that are parameterised with the coefficients of a

% discrete cosine function of time (P.v).

%--------------------------------------------------------------------------

M.v = @(T,P) spm_dctmtx(T,numel(P.C))*P.C(:);

% Having established the structure of the generative model we can now

% specify the prior expectations over the requisite parameters; including

% the overall rate at which the dynamics are expressed (P.t). Here, P.x

% parameterises the initial states at the beginning of the timeseries.

%--------------------------------------------------------------------------

pE.A = [10 16 1]; % parameters of pathophysiology

pE.B = [0 1 0;1 0 0;0 0 1]; % mapping to psychopathology

pE.C = [0 0 0 0 0 0 0 0]; % parameters of perturbations

pE.x = [16; 4; 32]; % initial physiological states

pE.u = [1,1,1]; % threshold for (three) outcomes

pE.t = 128; % time constant of dynamics

% The priors are completed by specifying the prior covariance, that

% determines which parameters are fixed and which are unknown and need to

% be estimated

%--------------------------------------------------------------------------

pC.A = spm_zeros(pE.A) + 1;

pC.A(1) = 0; pC.A(3) = 0; % allow only the 2nd param to vary

pC.B = spm_zeros(pE.B) + 1;

pC.B(3) = 0; pC.B(5) = 0; pC.B(7) = 0; % B([3, 5, 7]) are fixed based on previous experience

pC.C = spm_zeros(pE.C) + 1/64;

pC.x = spm_zeros(pE.x) + 1;

pC.u = spm_zeros(pE.u) + 0;

pC.t = 1;
